# Recurrent urethrovesical anastomotic strictures following artificial urinary sphincter implantation: a case report

**DOI:** 10.1186/1752-1947-6-94

**Published:** 2012-04-03

**Authors:** Ioannis Adamakis, Stavros I Tyritzis, Ioanna Vasileiou, Ioannis Katafigiotis, Ioannis Leotsakos, Sotiria Fergadaki, Konstantinos G Stravodimos, Constantinos A Constantinides

**Affiliations:** 1 Department of Urology, Athens University Medical School-LAIKO Hospital, Athens, Greece; 2 Department of Anesthesiology, LAIKO Hospital, Athens, Greece

## Abstract

**Introduction:**

The management of an anastomotic stricture after a radical prostatectomy can become a complex and difficult situation when an artificial urinary sphincter precedes the formation of the stricture. The urethral narrowing does not allow the passage of the routinely used urological instruments and no previous reports have suggested alternate approaches.

**Case presentation:**

We present the case of a 68-year-old Greek man diagnosed as having a recurrent anastomotic stricture approximately two years after a radical prostatectomy and three years after the implantation of an artificial urinary sphincter, and propose novel alternate methods of treatment. Our patient was first subjected to stricture incision with the use of a rigid ureteroscope with a holmium:yttrium-aluminium-garnet laser fiber, which was followed by a second successful attempt with the use of a pediatric resectoscope. After a one-year follow-up, our patient is doing well, with no evidence of recurrence.

**Conclusions:**

To the best of our knowledge, this is the first report of the management of recurrent urethral strictures following an artificial urinary sphincter implantation. Minimal invasive techniques with the use of small caliber instruments may offer efficient treatment options, diminishing the danger of urethral corrosion.

## Introduction

Despite improvements and refinements in the surgical techniques used for radical prostatectomy (RP), complications still exist. The commonest are incontinence and loss of erectile function. The next most common complication, with rates ranging from 0.48% to 32%, is the formation of urethrovesical anastomosis (UVA) stricture [1,2]. These strictures tend to have a high incidence of recurrence and several treatment options have been proposed such as dilatation, endoscopic cold-knife incision, urethral stent placement, electrocautery resection, anastomotic urethroplasty and intermittent self-catheterization. However, the problem becomes very complex in the presence of a previously placed artificial urinary sphincter (AUS). The approach to the stricture can be extremely difficult by the routinely used techniques and instruments. Until now, the management of recurrent contractures was simultaneous or before the placement of an AUS [3-5].

To the best of our knowledge, we present a case where novel methods were used to treat this complex and difficult situation.

## Case presentation

A 68-year-old Greek man was referred to our department for evaluation two years after an open retropubic RP. He presented with lower urinary tract symptoms and symptoms of urinary incontinence. His medical history was notable for hypertension and atrial fibrillation. Our patient was assessed with cystourethrography and cystourethroscopy and the presence of the anastomotic stricture was verified. An endoscopic cold-knife incision was performed successfully. Six months later, and after the recurrence of a urethral stricture was ruled out, our patient underwent an AUS placement for the management of incontinence. The decision to implant an AUS was taken after evaluating our patient with urethroscopy, during which a non-functioning external sphincter was observed. Our patient's post-operative course was uneventful. Our patient had regular follow-up visits with ultrasonography and was free of symptoms for a four-year period. Follow-up of our patient was performed with post-void residual and uroflow measurements. Three years after the implantation of the AUS, our patient was readmitted with voiding obstructive symptoms and the recurrence of the urethrovesical contracture was verified by urethroscopy. The AUS was deactivated at that time.

**Figure 1:**
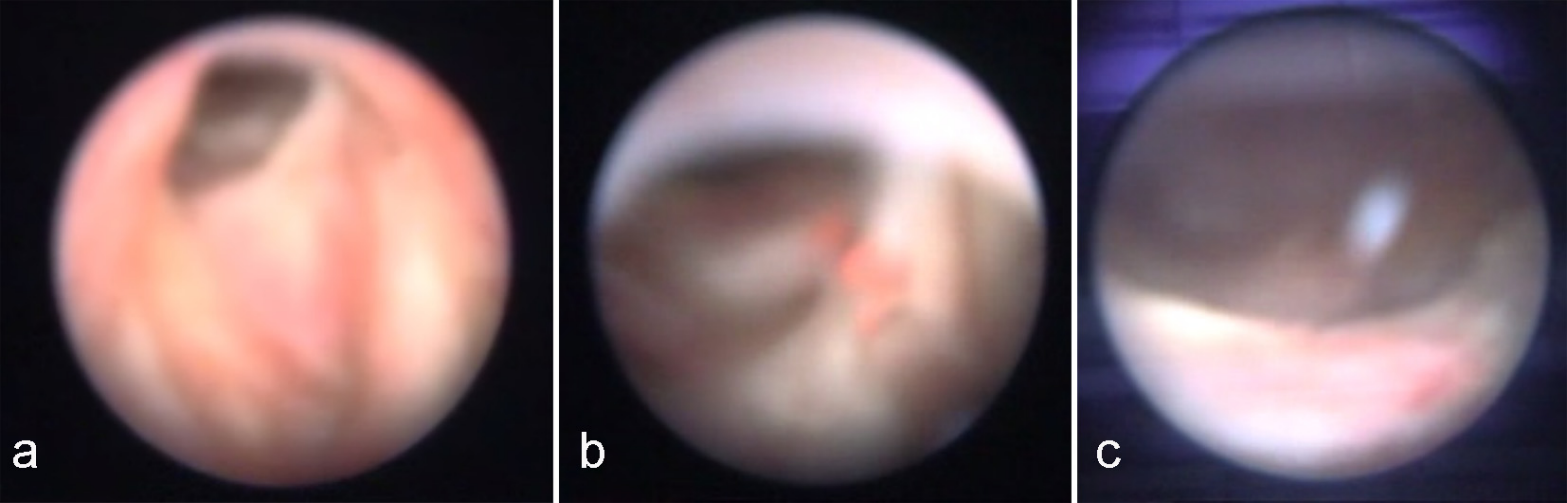
**Laser ablation of a stricture in the urethrovesical anastomosis**.

Under general anesthesia, with our patient in the lithotomy position, an 11F Olympus rigid ureteroscope was passed to the area of the stenosis (Figure 1). A holmium:yttrium-aluminium-garnet (Ho:YAG) laser with a 365 μm end-firing quartz fiber was passed through the working channel at a setting of 1J with a frequency of 10 Hz (10W). This could be increased during the procedure according to the surgeon's preference. Deep incisions in the scar tissue were performed by direct contact of the laser tip until visualization of the peri-vesical fat. An 18F Foley catheter was then introduced and left in place for three days.

**Figure 2:**
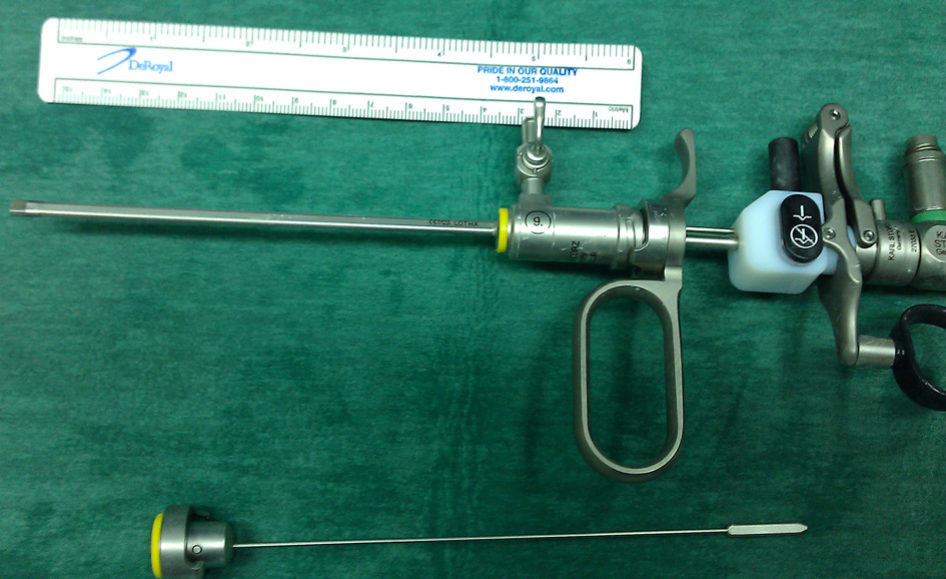
**Pediatric resectoscope**.

**Figure 3:**
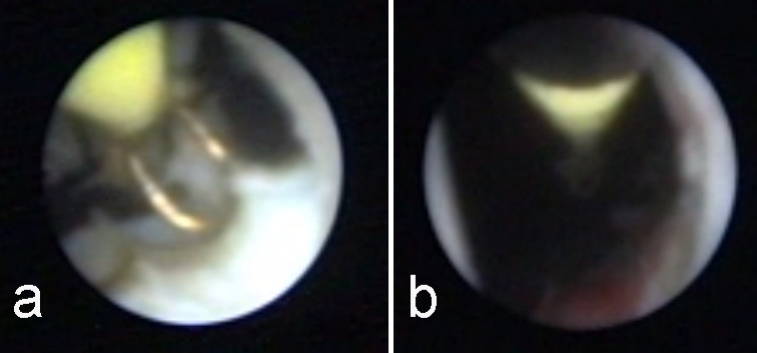
**Pediatric resectoscope during resection**.

Our patient again experienced a recurrence six months later. He was subjected to an endoscopic incision of the stricture with the use of a 9F pediatric resectoscope (Figure 2). Resection of the stricture was performed (Figure 3) and an 18F Foley catheter was placed. Our patient was discharged two days later after removal of the catheter and evaluation of his urinary function. Six weeks later, the AUS was reactivated. Our patient has been recurrence free after an 18-month follow-up period.

## Discussion

One of the concerns after RP is the occurrence of potentially recurrent UVA strictures. This complication appears normally within a few months following surgery. Risk factors for the occurrence of strictures are previous bladder neck surgery, urinary extravasation and excessive intra-operative blood loss [6,7]. There are varying degrees of association of anastomotic contracture and stress urinary incontinence [8,9]. The AUS was introduced as a treatment for post-prostatectomy incontinence with excellent results [7].

One of the major, but unresolved, concerns of AUS placement is the time of implantation following the initial management of the stricture. A period ranging from six weeks to seven months has been reported [4,10]. Because no conclusion had been made, we decided to wait for six months before we placed the AUS. Unfortunately, even this interval was not enough. Thus, prospective studies are needed to establish the optimal interval.

The management of a post-prostatectomy contracture has been performed with one-stage or two-stage procedures combining an aggressive incision of the stricture, followed by the AUS placement [4,5,7]. Others have suggested a transperineal urethroplasty combined with AUS implantation [11]. Although several treatment options such as dilatation, cold-knife incision, electrocautery incision or resection of the stenotic bladder neck, Urolume stent placement, triamcinolone injection and use of the Ho:YAG laser have been proposed, the optimal management of UVA contracture has not been determined yet. Also, no prospective studies have been published.

Yurkanin *et al. *[12] reported the achievement of good results by using cold- knife incision with a response rate of up to 87% after one session. A comparative study by Ramchandani *et al. *[13] however, reported that balloon dilatations were as effective as cold-knife incisions and suggested that cold-knife incisions should be left for complicated cases. In our case cold-knife incision seemed to be an attractive choice for the treatment of the UVA stenosis at our patient's first visit, since the stricture was detected early and the scarring process was still limited. Furthermore, the role of the transurethral incision remains highly debatable.

A two-stage approach with Urolume stenting of the contracture prior to an AUS has been reported with acceptable outcomes [3,10]. A recent study by the Baylor College of Medicine reported a 17-month satisfaction rate of 89% in nine patients [3]. Placement of a Urolume stent however, is not without complications, such as migration, hematuria, encrustation and re-obstruction due to the hyperplastic tissue ingrowth [3,14]. Moreover, the extraction of this stent can be very difficult for the urologist due to catastrophic effects to the urethral tissue.

An excellent review by Bader and colleagues has summarized Ho:YAG laser use [15]. The reviewed studies were neither randomized nor prospective, and the patient cohorts and the follow-up periods were limited [4,16,17]. The Ho:YAG laser is safe and easy to handle and was reported to have a success rate of 83% in a series of 24 patients [4]. Under direct vision a controlled incision and vaporizing of the scar tissue can be performed [18]. The end-firing fiber of the holmium laser is light and flexible and can be used with a rigid and a flexible endoscope, due to its small caliber. Although the physical characteristics of this laser type are advantageous, due to minimal tissue penetration and accurate targeting, safe conclusions about its efficacy and effectiveness cannot be drawn.

All of the above approaches cannot be safely used in the presence of an AUS due to urethral narrowing. In our patient the passage to the area of the stenosis was difficult. Thus, the use of the 11F rigid ureteroscope along with the Ho:YAG laser seemed to be the ideal treatment for our patient since the flexible end-firing fiber of the laser made the access to the stricture easier. Furthermore, with the holmium laser we could control the firing pulses accurately with a foot switch; thus damage to the collateral healthy tissue was prevented [14], which is very important especially in a patient with an AUS who presents with a recurrent UVA. In general, instrumentation to the urethra in such patients could lead to urethral erosion, subsequent AUS removal and all the relevant repercussions for the patient.

In an effort to minimize the danger of erosion, minimally invasive techniques are required. An interesting approach was reported by Eltahawy *et al. *using a pediatric cystoscope [4]. The small caliber of this scope (7.5F) is ideal for passing through a narrowed urethra. However, we decided to try a pediatric resectoscope (9F) (Figure 1) due to our previous failure with the Ho:YAG laser. The intra-operative use of the resectoscope was excellent, allowing for a potent recanalization. Two important issues should be mentioned: the first is related to the movement of the resectoscope, which is passive. The second one concerns the resected chips, which are easily removed by irrigation saline via the working channel of the resectoscope due to their small size. We advocate the presence of a pediatric scope in an adult urological department, despite its cost, because it can be life saving in cases of urethral stenosis in general.

## Conclusions

Patients who are post-RP with an implanted AUS with the complication of an UVA contracture can be difficult to manage due to narrowing of the urethra. Although the ideal treatment for recurrent UVA strictures remains debatable, our case shows that the urologist must be aware of several treatment options, especially when a plethora of instruments are available. Use of a rigid ureteroscope or the pediatric resectoscope seems appealing due to their small caliber, but larger patient series and longer follow-up periods are essential in order to draw safe conclusions.

## Consent

Written informed consent was obtained from the patient for publication of this manuscript and any accompanying images. A copy of the written consent is available for review by the Editor-in-Chief of this journal.

## Competing interests

The authors declare that they have no competing interests.

## Authors' contributions

ST and IV drafted the manuscript and gathered patient data. ST, IA, KG analyzed and interpreted the data from our patient. IL, IK and SF followed up our patient and recorded the outcomes. IA performed the surgical operations, assisted by ST, and supervised the writing of the manuscript. CA supervised the writing of the manuscript and made critical revisions. All authors read and approved the final manuscript.
